# Pregnancy-induced effects on memory B-cell development in multiple sclerosis

**DOI:** 10.1038/s41598-021-91655-9

**Published:** 2021-06-09

**Authors:** Malou Janssen, Liza Rijvers, Steven C. Koetzier, Annet F. Wierenga-Wolf, Marie-José Melief, Jamie van Langelaar, Tessel F. Runia, Christianne J. M. de Groot, Rinze Neuteboom, Joost Smolders, Marvin M. van Luijn

**Affiliations:** 1 Department of Immunology, Erasmus MC, Rotterdam, The Netherlands; 2 Department of Neurology, Erasmus MC, Rotterdam, The Netherlands; 3 MS Center ErasMS, Erasmus MC, Rotterdam, The Netherlands; 4 Department of Obstetrics and Gynaecology, Amsterdam UMC, Vrije Universiteit Amsterdam, VU Medical Center, Amsterdam, The Netherlands

**Keywords:** Immunology, Neurology

## Abstract

In MS, pathogenic memory B cells infiltrate the brain and develop into antibody-secreting cells. Chemokine receptors not only define their brain-infiltrating capacity, but also assist in their maturation in germinal centers. How this corresponds to pregnancy, as a naturally occurring modifier of MS, is underexplored. Here, we aimed to study the impact of pregnancy on both ex vivo and in vitro B-cell differentiation in MS. The composition and outgrowth of peripheral B cells were compared between 19 MS pregnant patients and 12 healthy controls during the third trimester of pregnancy (low relapse risk) and postpartum (high relapse risk). Transitional, and not naive mature, B-cell frequencies were found to drop in the third trimester, which was most prominent in patients who experienced a pre-pregnancy relapse. Early after delivery, these frequencies raised again, while memory B -cell frequencies modestly declined. CXCR4 was downregulated and CXCR5, CXCR3 and CCR6 were upregulated on postpartum memory B cells, implying enhanced recruitment into germinal center light zones for interaction with T follicular helper (T_FH_) cells. Postpartum memory B cells of MS patients expressed higher levels of CCR6 and preferentially developed into plasma cells under T_FH_-like in vitro conditions. These findings imply that memory B- cell differentiation contributes to postpartum relapse risk in MS.

## Introduction

MS is a chronic inflammatory and demyelinating disease of the CNS, for which a key role for B cells in the pathogenesis has been shown in recent years^1^. In the relapsing phase of MS, naive B cells escape peripheral tolerance checkpoints^2^ and develop into memory populations that activate CNS-infiltrating, IFN-γ-producing CD4^+^ T cells^3^. This is further supported by the reduction in T-cell activation in MS patients after receiving anti-CD20 therapy^4^. Normally, memory B cells interact with T cells within germinal centers (GCs) of peripheral secondary lymphoid organs. Our group recently demonstrated that B cells utilize IFN-γ to drive their CXCR3-expression, boosting their ability to recruit to MS brain tissue as well^5^. Hence, in MS, CNS-infiltrating memory B cells are probably reactivated by T cells in perivascular spaces^6^ and ectopic lymphoid structures in the cerebral meninges^7^ to further mature into plasmablasts/plasma cells and contribute to focal inflammation^6^.

IFN-γ-induced CXCR3 expression on B cells is not only associated with increased homing to inflamed tissue and the formation of ectopic lymphoid structures, but also with aberrant plasma cell differentiation within GCs^8,9^. This is further controlled by the expression of other chemokine receptors, such as CXCR4, CXCR5 and CCR6. CXCR4 and CXCR5 orchestrate the GC response by guiding B cells into dark and light zones respectively. Centroblasts undergo several rounds of proliferation and somatic hypermutation in the dark zone, while centrocytes interact with follicular dendritic cells and T follicular helper (T_FH_) cells to undergo antigen-specific selection in the light zone^10^. CCR6 has been reported to promote their development and antigen responsiveness in the light zone^11,12^. Therefore, specific aberrancies in chemokine receptor profiles may affect the outgrowth of B cells into memory and plasmablasts/plasma cells^13^. Currently, it remains to be determined whether this is related to relapse risk in MS.

Notably, in pregnant MS patients, relapse risk is reduced by approximately 70% in the third trimester. This increases in the first three months after delivery, with almost 30% of patients having a postpartum relapse^14^. Although current diagnostic and treatment strategies probably contribute to an attenuation of this fluctuation^15^, these specific phases of pregnancy can still be considered periods of relatively low and high relapse risk in MS. Pregnancy is known to cause a shift from T_H_1 to T_H_2 responses^16^ and promote the expansion of circulating T_FH_ cells^17^, therefore likely affecting the maturation of B cells in GCs.

In this experimental study, our primary aim was to determine the effects of pregnancy as naturally occurring disease modifier on peripheral B-cell differentiation in MS. We assessed the frequencies and GC-related chemokine receptor profiles on ex vivo B-cell subsets in paired first trimester, third trimester and early postpartum blood samples of MS patients and healthy controls. In addition to this, we used a T_FH_-like cell culture system to investigate how in vitro memory B-cell differentiation into plasmablasts or plasma cells differs between periods of relatively low (third trimester) and high (early postpartum) relapse risk.

## Methods

### Participants

Nineteen pregnant women with RRMS were included at the MS Center ErasMS as part of our previous study^18^ and retrospectively validated to match the most recent McDonald 2017 criteria^19^. Patients did not use any immune modulatory medication before, during and early after pregnancy. Two patients gave birth via a cesarean section and all others had vaginal deliveries. No relapses were observed during pregnancy. During the early postpartum period, 6 patients developed a clinically defined relapse^20^. These patients did not differ from non-relapsing patients in their third trimester expanded disability status scale (EDSS) scores. No MRI evaluations for disease activity were performed. Additionally, 12 age-matched healthy pregnant women were included, who did not have central nervous system or inflammatory disease and were seen at the outpatient obstetric clinic at Erasmus MC. None of the healthy pregnant women used immunomodulatory medication before or during the study, were hypertensive, experienced recurrent abortions or were diagnosed with diabetes mellitus. Clinical characteristics of patients and controls are depicted in Table 1. All participants gave written informed consent, this study was approved by the medical ethics committee of Erasmus MC and all methods were performed in accordance with the relevant guidelines and regulations.

**Table 1:** Clinical information of pregnant MS patients and healthy controls.

	Ex vivo		
	RRMS, no PP relapse	RRMS, PP relapse	HC
Number of individuals	13	6	12
Median maternal age (range)	33.3 (26.8–33.8)	32.1 (29.8–33.7)	33.3 (27.7–34.1)
Median EDSS third trimester (range)	1.5 (0.0–2.0)	1.0 (0.0–2.0)	N.A
Median EDSS postpartum (range)	1.0 (0.0–2.0)	1.8 (1.0–4.5)	N.A
Nullipara	4	1	9
Caesarean section	2	0	1
(Pre)eclampsia	0	0	0
Median gestation (weeks, range)	40 (39.0–41.0)	38.0 (38.0–39.0)	39.0 (37.0–40.0)

	In vitro		
	RRMS, no PP relapse	RRMS, PP relapse	HC
Number of individuals	8	3	5
Median maternal age	31.9 (26.8–37.3)	31.5 (N.A)	27.9 (27.2–27.9)
Median EDSS third trimester (range)	1.5 (0.0–2.0)	2.0 (0.0–2.0)	N.A
Median EDSS postpartum (range)	1.0 (0.0–2.0)	2.0 (1.5–3)	N.A
Nullipara	5	2	1
Caesarean section	2	0	0
(Pre)eclampsia	0	0	0
Median gestation (weeks, range)	39.5 (38.0–41.0)	39 (38.0–40.0)	38.6 (34.0–42.0)

Information on cesarean section, nullipara, pre(eclampsia) and gestational period was missing for 1 RRMS patient with a PP relapse.

*PP* postpartum.

### PBMC isolation, flow cytometry and antibodies

PBMCs from patients and controls were collected in the first and third trimester of pregnancy as well as 4–8 weeks after delivery (postpartum). For sample collection, we used Vacutainer CPT^®^ tubes containing sodium heparin according to manufacturer’s instructions (BD Biosciences, Erembodegem, Belgium). After isolation, cells were taken up in RPMI 1640 (Lonza, Basel, Switzerland) containing 20% fetal calf serum (Lonza) and 10% dimethyl sulfoxide (Sigma-Aldrich, St Louis, MO) and stored in liquid nitrogen until further use. Cells were pre-incubated with Fixable Viability Stain 700 (BD Biosciences) for 15 min at 4 °C. The following monoclonal antibodies were used stained for 30 min at 4 °C: CD27 (BV421, M-T271), CD138 (BV605 and PE-CF594, MI15), CXCR4 (PE-CF594, 12G5), CXCR5 (PercCP, RF8B2), IgD (PE-CF594, IA6), IgG (APC-H7, G18-145; all BD Biosciences), CCR6 (PE, G034E3), CD19 (BV785, HIB19), CD38 (BV605 and PE-Cy7, HIT2), CXCR3 (APC and PE-Cy7, G025H7), CXCR4 (APC-Cy7, 12G5) and IgM (BV510, MHM-88; all Biolegend, London, UK). Stained cells were measured using an LSRII‐Fortessa flow cytometer. For in vitro culture experiments, memory (CD19^+^CD3^-^CD27^+^) B cells were purified using a FACSAria III sorter. Data were analyzed using FACS Diva software, version 8.0.1 (all BD Biosciences). Since ex vivo B-cell phenotyping of the relapsing MS group was performed in a separate study, it was not possible to compare the MFI of functional markers with the non-relapsing MS or control groups.

### Germinal center-like B-cell differentiation assay

In vitro B-cell differentiation assays were performed as recently described^5,21^. In short, irradiated murine 3T3 fibroblasts expressing human CD40L were co-cultured with sorted memory B cells in the presence of IL-21 (50 ng/ml; Thermo Fisher Scientific, Landsmeer, The Netherlands). After 6 days of culturing, viable CD19^+^ cells were analyzed using flow cytometry and supernatants were collected and stored at -80 °C until further use.

### IgM and IgG ELISA

IgM and IgG levels were determined in supernatants of memory B cells cultured for 6 days using ELISA. After overnight coating with goat anti-human Ig (1 mg/ml; Southern Biotech, Birmingham, USA) at 4 °C, flat-bottom 96-well plates (Corning, Tewksbury, USA) were washed with PBS/0.05% Tween-20 and subsequently blocked with PBS/5%FCS for 2 h at RT. Samples were added for 1.5 h at room temperature. After washing, peroxidase-conjugated goat anti-human IgG (Thermo Fisher Scientific) or rabbit anti-human IgM (Jackson, Uden, The Netherlands) were used to detect bound antibody. 3,3′,5,5′-Tetramethylbenzidine substrate (Thermo Fisher Scientific) was used to reveal peroxidase activity. Reactions were stopped with sulfuric acid and optical densities were measured at 450 nm using a BioTek Synergy 2 reader (Winooski, USA). Concentrations were calculated using standard curves for IgM and IgG.

### Statistical analysis

Graphpad Prism software (version 8) was used for statistical analyses. Both percentages and mean fluorescent intensity (MFI) are shown as individual data points together with the corresponding mean. We compared paired data using Wilcoxon signed-rank tests and data between clinical groups using 2-way analysis of variance (ANOVA) with Bonferroni’s multiple comparison tests, unless stated otherwise. *P* values < 0.05 were considered statistically significant.

## Results

Our overall aim was to explore how pregnancy as naturally occurring disease modifier influences the peripheral B-cell lineage in MS. To properly address the impact of pregnancy without relapses as a potential confounder, we first analyzed ex vivo B-cell differentiation profiles in paired first trimester (T1), third trimester (T3) and early postpartum (PP) blood samples from 13 MS patients without a postpartum relapse. These profiles were compared with blood samples from the same periods of 12 healthy controls (Table 1)^18^ to determine their relation to MS. Finally, we verified whether relapses are associated with alterations in B-cell profile and compared B-cell development into antibody-secreting cells in vitro for samples from low and high relapse risk periods (T3 and PP, respectively).

### Pregnancy alters the peripheral B-cell compartment resulting in memory populations with increased Ig expression in the early postpartum period

**Figure 1:**
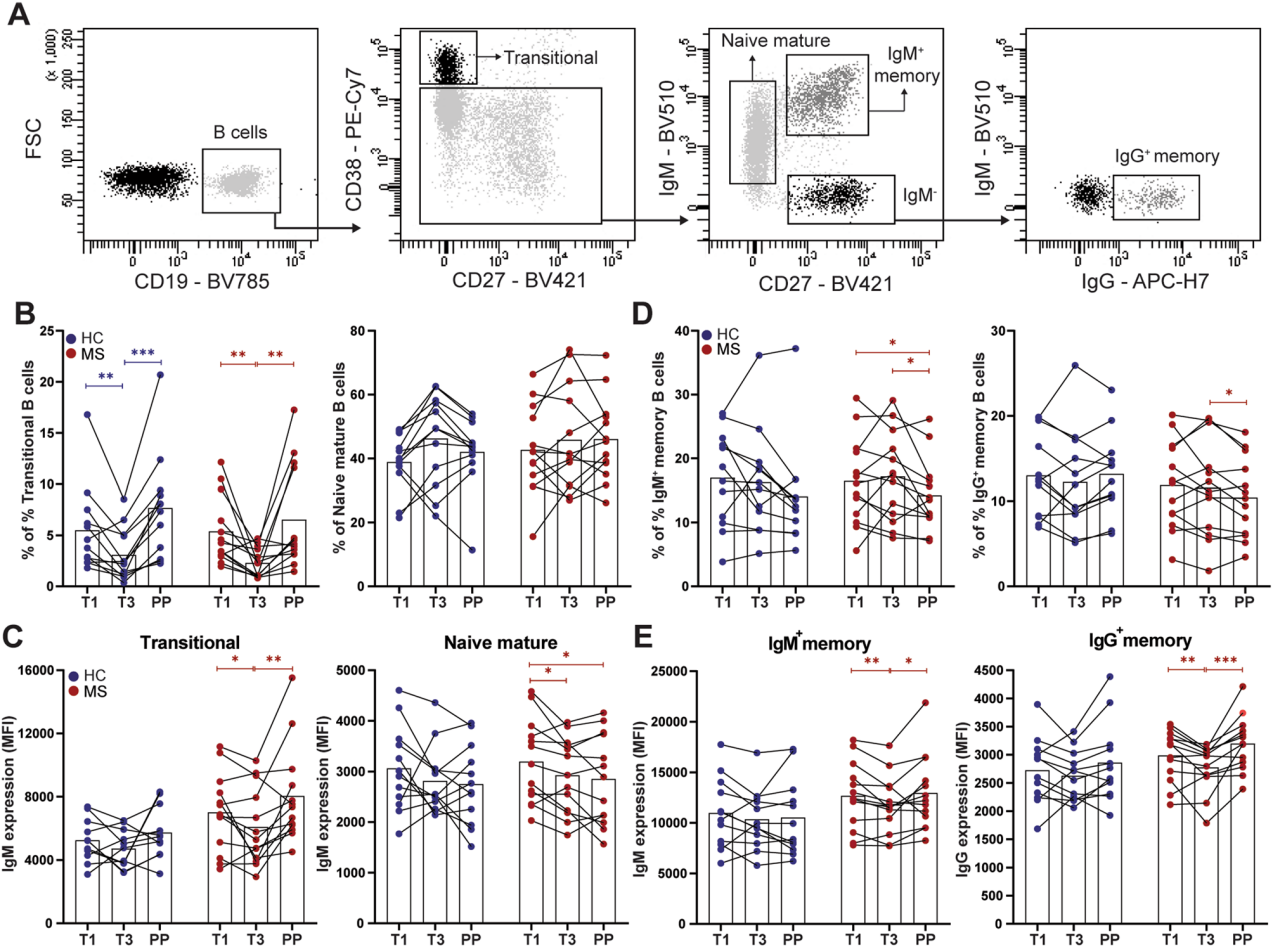
Frequencies of circulating naive and memory B-cell subsets during and early after pregnancy in MS patients and healthy controls. (**A**) Representative gating of viable CD19^+^ B-cell subsets: transitional (CD38^high^CD27^-^), naive mature (CD38^-/dim^CD27^-^IgM^+^), IgM memory (IgM^+^CD27^+^) and IgG memory (IgG^+^CD27^+^) B cells. The percentages of transitional and naive mature B cells (**B**) as well as IgM and IgD expression (MFI) on these subsets (**C**) were compared between paired first trimester (T1), third trimester (T3) and postpartum (PP) blood samples of 13 MS patients who did not experience a postpartum relapse (red) and 12 HC (blue). Similar analyses were performed for IgM^+^ and IgG^+^ memory B cells (**D**,**E**). Wilcoxon signed-rank test was performed to compared the different gestational periods. * p < 0.05, ** p < 0.01, *** p < 0.001.

We analyzed the proportions of different naive and memory populations including transitional (CD38^high^CD27^-^), naive mature (CD38^-/dim^CD27^-^IgM^+^) as well as IgM^+^ and IgG^+^ memory (CD38^-/dim^CD27^+^) B cells using flow cytometry (Fig. 1A). In MS patients without a postpartum relapse (n = 13) and healthy controls, the proportion of transitional B cells declined from first to third trimester and recuperated after delivery (Fig. 1B). Naive mature B-cell frequencies were not different between periods, resulting in elevated naive mature/transitional B-cell ratios per individual in the third trimester (Supplementary Fig. 1A). These ratios were further increased in patients with a relapse one year before pregnancy (Supplementary Fig. 1B) and were lower in MS patients with an early postpartum relapse (n = 6; Table 1 and Supplementary Fig. 1C). IgM and not IgD expression was significantly increased on postpartum transitional B cells, which was not seen for naive mature B cells and was the most pronounced in MS patients (p = 0.0056, Fig. 1C and Supplementary Fig. 1D). Both IgM^+^ and IgG^+^ memory B cells showed a moderate decline in frequencies, but a significant increase in Ig surface expression in the postpartum period (p = 0.0473 for IgM^+^ and p = 0.0010 for IgG^+^; Fig. 1D,E). These differences in expression level and memory fractions were not influenced by an early pre-pregnancy and postpartum relapse (Supplementary Fig. 2).

The pregnancy-induced disturbances in naive to memory B-cell development ex vivo may imply favored differentiation in GCs in the early postpartum period^22^.

### Memory B cells reveal a more GC light zone-related chemokine receptor expression profile in postpartum versus third trimester samples

**Figure 2:**
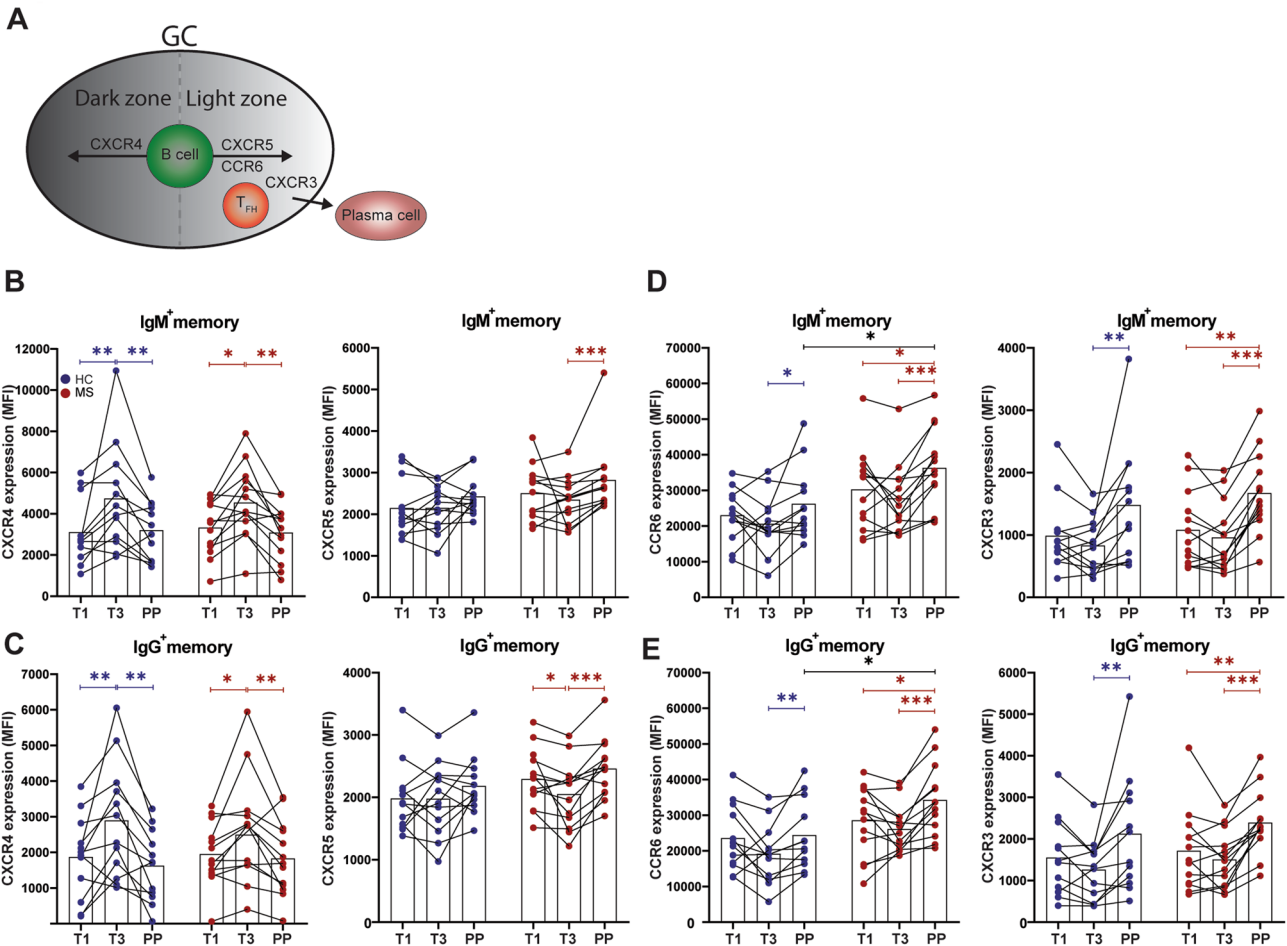
Chemokine receptor expression on circulating memory B cells during and early after pregnancy in MS patients and healthy controls. (**A**) Schematic display of chemokine receptors involved in GC-dependent organization and maturation of B cells. Expression of dark zone-associated CXCR4 and light zone-associated CXCR5, CCR6 and CXCR3 was compared between IgM^+^ (**B,D**) and IgG^+^ (**C,E**) memory B cells from first trimester (T1), third trimester (T3) and postpartum (PP) samples from 13 MS patients without a postpartum relapse (red) and 12 HC (blue). Wilcoxon signed-rank test was performed to compare the different gestational periods. Two-way ANOVA with Bonferroni’s multiple comparison test was performed to compare HC with MS patients. * p < 0.05, ** p < 0.01, *** p < 0.001, **** p < 0.0001.

Chemokine receptors selectively regulate GC organization and maturation of B cells (Fig. 2A). First trimester, third trimester and postpartum B cells were analyzed for the expression of CXCR4 and CXCR5, which mediate dark and light zone localization, respectively^10^, as well as CCR6 and CXCR3, which contribute to memory recall and antibody responses^8,23^, respectively.

CXCR4 was downregulated, while CXCR5 was upregulated on postpartum versus third trimester B cells. This was seen for both memory (IgM^+^ and IgG^+^; Fig. 2B,C) and naive (Supplementary Fig. 3A) B cells. The postpartum rise in CXCR5 was significant in the MS but not in the healthy control group. CXCR4 and CXCR5 levels were higher on naive than memory B cells (Supplementary Fig. 4A). CCR6 and CXCR3 were upregulated on postpartum B cells in both groups (Fig. 2D,E; Supplementary Fig. 3B). In the MS group, postpartum CCR6 levels were higher compared to healthy controls as well as first trimester B cells (Fig. 2D,E). For all the groups and periods, CCR6 was predominantly expressed on naive B cells, while CXCR3 expression was most pronounced on memory B cells (Supplementary Fig. 4B). In contrast to CXCR3, CCR6 was not upregulated on postpartum B cells from 4 of 6 MS patients experiencing a postpartum relapse (Supplementary Fig. 5). We found no significant impact of a pre-pregnancy relapse on chemokine receptor expression (data not shown).

Together with the increased Ig surface levels (Fig. 1E), the distinct chemokine receptor expression profile for ex vivo postpartum memory B cells particularly from MS patients (CXCR3^high^CXCR4^low^CXCR5^high^CCR6^high^) implies an increased potential of these cells to recruit and further develop in the GC light zone.

### Postpartum memory B cells of MS patients preferentially develop into Ig-secreting plasma cells under T_FH_-like conditions in vitro

**Figure 3:**
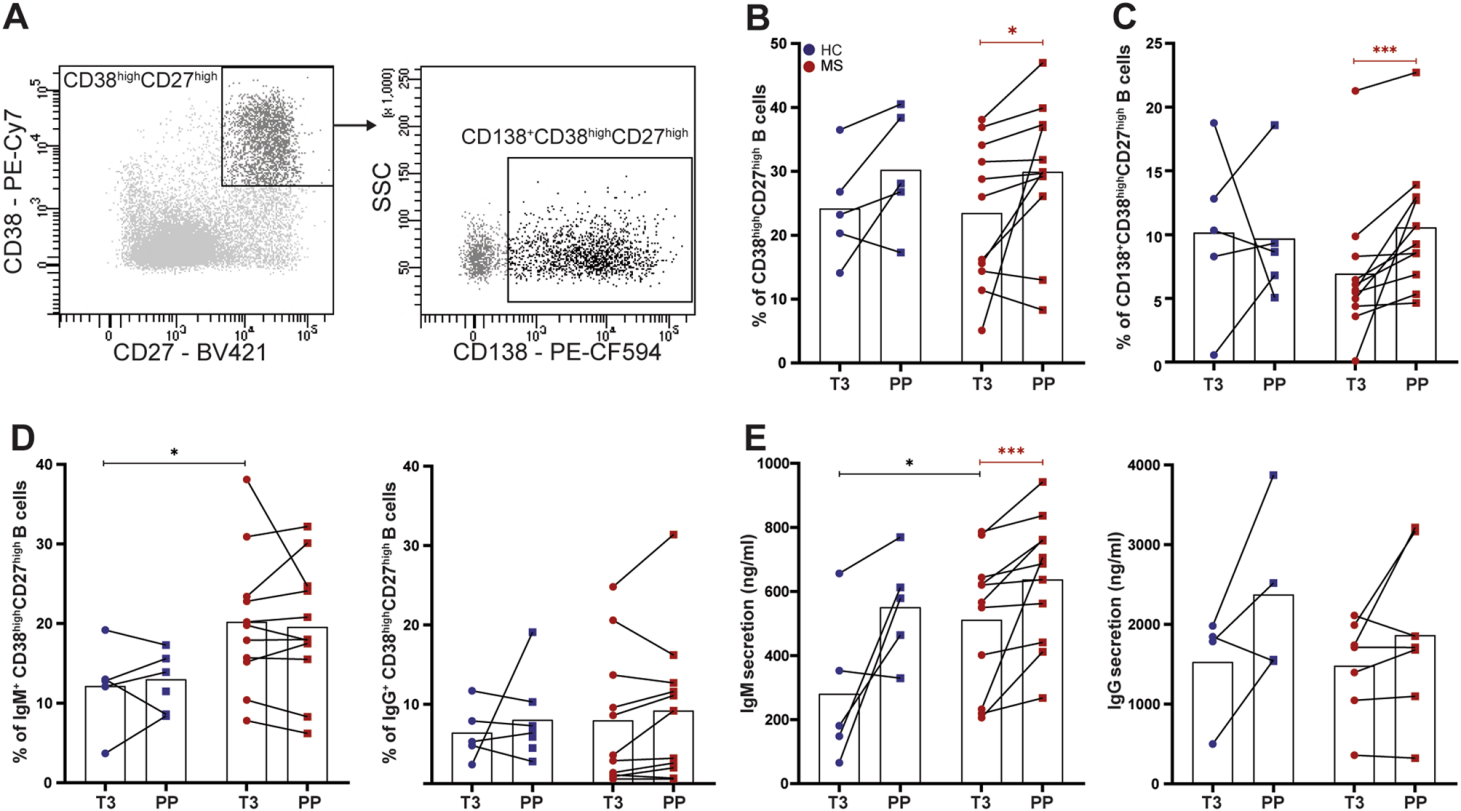
In vitro outgrowth of antibody-secreting cells using memory B cells from paired third trimester and postpartum blood samples. (**A**) Representative gating of CD138-expressing antibody-secreting cells (CD38^high^CD27^high^) after culturing of memory B cells under IL-21/CD40L-stimulating (T_FH_-like) conditions for 6 days. Fractions of CD38^high^CD27^high^ plasmablasts/plasma cells (**B**), CD138^+^CD38^high^CD27^high^ plasma cells (**C**) and IgM^+^ and IgG^+^ CD38^high^CD27^high^ plasmablasts (**D**; FACS), as well as IgM and IgG secretion (**E**; ELISA) were compared between cultures with third trimester (T3) and postpartum (PP) memory B cells from 11 MS patients (red) and 5 HC (blue). Wilcoxon signed-rank test was performed to compare third trimester and postpartum samples. Two-way ANOVA was performed to compare MS and HC groups. * p < 0.05, *** p < 0.001.

B-cell memory formation is generated through the help of T_FH_ cells within the GC light zone. After reaching the CNS, memory B cells are likely reactivated to develop into potent antibody-secreting cells in MS patients. To assess whether B cells are prone for such recall responses in the high-risk postpartum period in MS, memory (CD27^+^) B cells were purified from paired third trimester and postpartum samples of 11 MS patients and compared for their outgrowth into plasmablasts/plasma cells in vitro. Under IL-21- and CD40L-inducing conditions, which mimic a T_FH_ cell response, more antibody-secreting cells (CD38^high^CD27^high^) were formed in cultures with postpartum versus third trimester memory B cells (p = 0.023, Fig. 3A,B). A similar trend was seen for memory B cells derived from paired samples of healthy controls (Fig. 3B). When we analyzed plasma cell (CD138^+^CD38^high^CD27^high^) frequencies, these were mainly increased for postpartum memory B cells from MS patients, which were significantly higher than those from healthy controls (p = 0.001, Fig. 3C). For the MS group, IgM^+^ and not IgG^+^ plasmablasts (CD38^high^CD27^high^) were more induced in both third trimester and postpartum memory B-cell cultures (Fig. 3D). Especially IgM levels were elevated in the supernatants of postpartum memory B-cell cultures, but were not different between patients and healthy controls (Fig. 3E).

These results show that memory B cells are highly capable of differentiating into antibody-secreting cells early after parturition, which may contribute to the high postpartum relapse risk in MS.

## Discussion

Pregnancy causes a transient period of immune suppression, which is lost early after delivery in MS. Here, we studied the functional impact of pregnancy as a disease modifier on peripheral B-cell subsets (primary aim), whether this is differentially regulated between MS patients and healthy controls (secondary aim) and if this is affected by a pre- or post-pregnancy relapse (tertiary aim). The chemokine receptor expression pattern (CXCR3^high^CXCR4^low^CXCR5^high^CCR6^high^) found for ex vivo postpartum B cells implied a marked propensity to recruit to the T_FH_ cell-containing GC light zone^10,11^. The elevated expression of CCR6 on ex vivo postpartum memory B cells of MS compared to healthy females pointed toward increased, T cell-dependent recall responsiveness^11,23^. This was supported by their ability to differentiate into plasma cells under T_FH_-like conditions in vitro.

Regarding naive B cells, the proportion of circulating transitional B cells was found to be decreased in third trimester compared to postpartum samples. A similar observation was made in a previous study^24^. We additionally show that this decline occurs in both MS patients and healthy controls and is also seen when comparing to samples from the first trimester, a phase in which relapse rates are less reduced^14^. These pregnancy-induced alterations in B lymphopoiesis are probably the result of increased hormone levels in the third trimester, keeping transitional B cells in check due to a lack of multidrug resistance receptor 1 (MDR1)^25^, a glycoprotein which pumps steroids out of cells. We recently found a similar impact of steroid treatment in patients with MS, AQP4-IgG^+^ neuromyelitis optica spectrum disorder and MOG-IgG-associated disease^26^. Because of the rise in IgM^high^ transitional B cells early after delivery, one could speculate that this results in increased entrance of potentially autoreactive naive mature B cells to GCs, resulting in the development of pathogenic memory subsets that are destined to enter the CNS and contribute to an MS relapse. Although we did not touch upon their CNS-infiltrating ability in this study, we can at least assume that the postpartum increase in CXCR3 expression mediates local B-cell enrichment in MS^5^. This may be further induced by the observed abundance of CCR6 on postpartum B cells of MS patients.

The postpartum upregulation of CXCR5 especially seen in MS patients is likely involved in B-cell organization rather than recruitment in the CNS^10,27^. Our group previously reported an upregulation of CXCR4 during MS onset in non-pregnant patients^28^. The observed reduction in CXCR4 expression during the high relapse risk postpartum period is therefore counterintuitive. A possible explanation for this discrepancy may be that during a primary response, CXCR4^high^ naive mature B cells escape from T_FH_-mediated selection in the GC light zones of peripheral secondary lymphoid organs^13^, while CXCR4^low^ memory B cells are more prone to interact with T_FH_ cells and develop into long-lived plasma cells during a recall response in the CNS. CCR6 could facilitate such recall responses^23^ and, together with CXCR3, has also been associated with the production of high affinity antibodies^29^.

Thus far, the potential of functionally distinct memory B cells to develop into plasma cells has been relatively understudied in MS. Despite the current focus on antibody-independent B-cell functions, this is of high relevance as long-lived plasma cells reside within the chronically inflamed CNS of MS patients^30^. The clinical relevance of local Ig production has become apparent from the increased risk of CIS to MS conversion in patients with CSF oligoclonal bands^31^, which are present in more than 95% of MS patients and indicates ongoing IgG production in the CNS. Consistently, the absence of B cells in brain lesions of MS patients is associated with a lack of CSF oligoclonal bands, a lower intrathecal IgG production, and a more favorable outcome^32^. The observation that anti-CD20 treatment reduces CSF B cell numbers while oligoclonal bands persist^33^ suggests that intrathecal IgG are mainly produced by (CD20^-^) long-lived plasma cells in the CNS. Recently, it has been shown that MS myelin is bound by IgG and that IgG immune complexes trigger human microglia, resulting in enhanced production of pro-inflammatory cytokines^34^.

Our study has some limitations. The relatively low numbers of included subjects hampered the analysis of MS risk groups based on disease activity before and after pregnancy. Sequential data collection of such patients is difficult, but the accumulating evidence for the safe continuation during pregnancy of various disease modifying therapies may increase options^35^. Furthermore, we did not perform in vitro B-cell cultures with pregnancy-related sera or hormones to verify whether the observed GC light zone phenotype is controlled by extrinsic factors. Finally, although beyond the scope of this study, intrinsic factors such as memory B-cell EBV load could contribute to the increased CXCR3 expression and plasma cell formation^21^ as seen early after delivery.

Together, this work provides new insights into how B-cell development is affected during high and low risk periods associated with pregnancy in MS. We demonstrate a first link between chemokine receptor expression profiles and the capacity of (potentially brain-infiltrating) memory B cells to differentiate into plasma cells, which should be further studied in the near future. This may not only help to decipher underlying mechanisms of local B-cell accumulation and antibody production, but also offer new tools to better predict disease activity in patients with MS.

## Supplementary Information

Supplementary Figures.

## Data Availability

The data supporting the findings of this study are available from the corresponding author upon reasonable request.
