# Supplementary material for: Pregnancy-induced effects on memory B-cell development in multiple sclerosis
Source: Sci Rep. 2021 Jun 9;11:12126. doi: 10.1038/s41598-021-91655-9 (PMC8190290; doi:10.1038/s41598-021-91655-9)
Supplement: Supplementary file 1 — Supplementary Figures. [file 41598_2021_91655_MOESM1_ESM.docx]

**
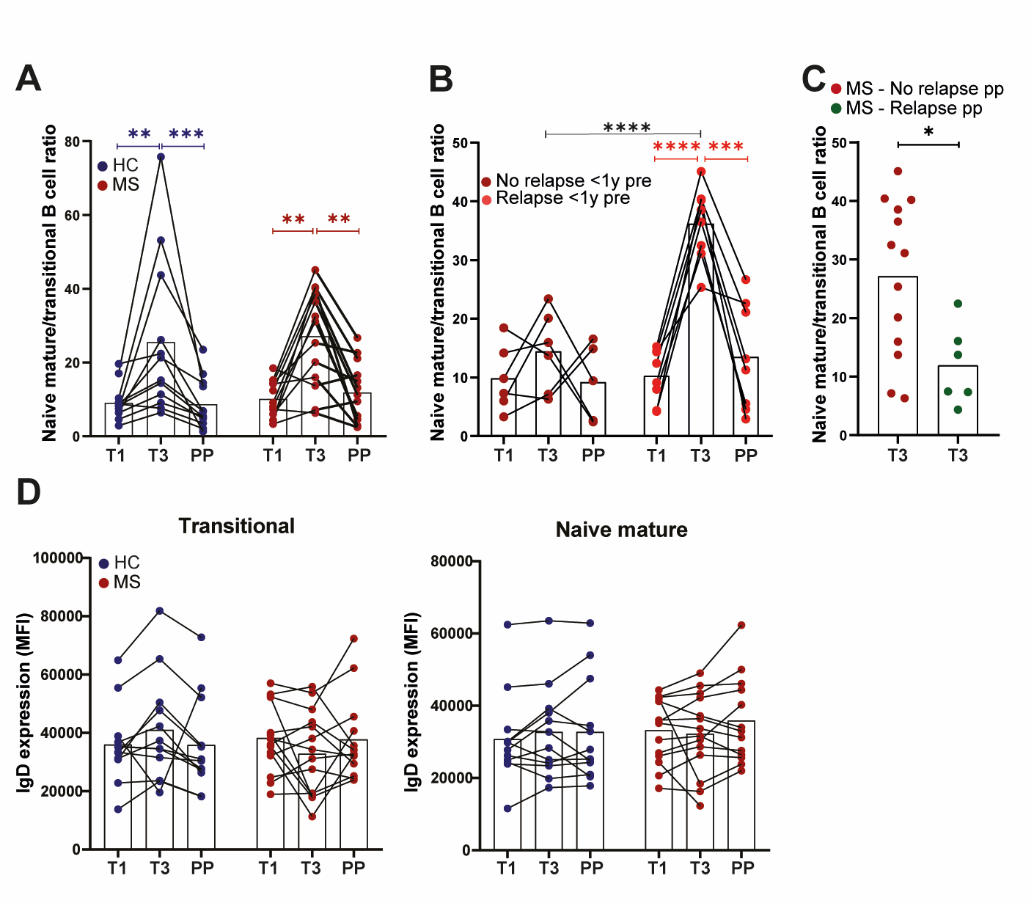
**

**Supplementary figure 1: The naive mature/transitional B cell ratio and the IgD surface expression on these subsets**

(**A**) The naive mature/transitional B cell ratio in the different gestational periods of 12 HC (blue) and 13 MS patients (red), which in **(B)** were separated into MS patients with (n = 8) or without (n = 6) a relapse within 1 year before pregnancy. **(C)** The naive mature/transitional B cell ratio in the 3^rd^ trimester of MS patients with (n=6) or without (n=13) a postpartum relapse. **(D)** IgD expression on transitional and naive mature B cells was compared between paired first trimester (T1), third trimester (T3) and early postpartum (PP) samples of 12 HC (blue) and 13 MS patients without a postpartum relapse (red). Wilcoxon signed-rank test was performed to compare the different gestational periods. Mann-Whitney U test was performed in **C**. * p<0.05, ** p<0.01, *** p<0.001, **** p<0.0001.

**
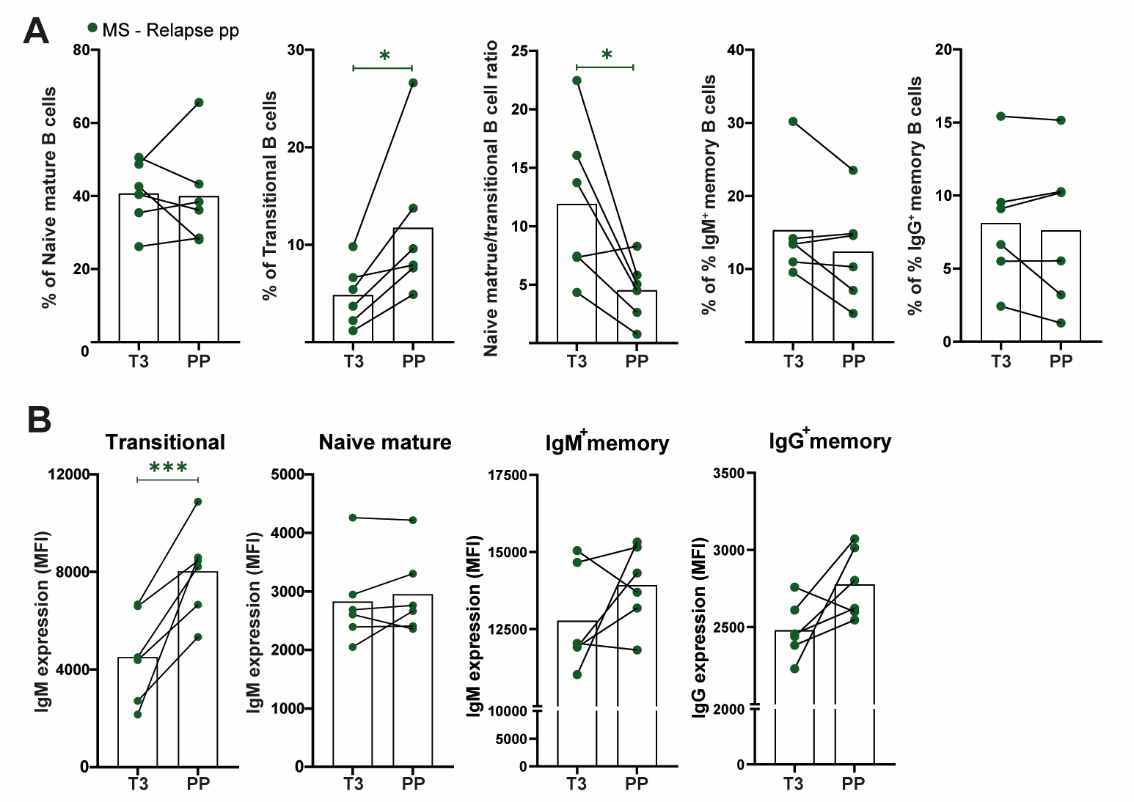
**

**Supplementary figure 2: Frequencies of B cell subsets and immunoglobulin expression in MS patients with a postpartum relapse**

**(A)** The percentage of transitional and naive mature B cells of third trimester (T3) and early postpartum (PP) periods in MS patients with a post-pregnancy relapse (n = 6), as well as their ratio and the frequency of IgM^+^ and IgG^+^ memory B cells. (**B**) Immunoglobulin expression on memory B cells of MS patients with a postpartum relapse (n = 6). Wilcoxon signed-rank test was performed to compare the different gestational periods. * p<0.05, *** p<0.001.

**
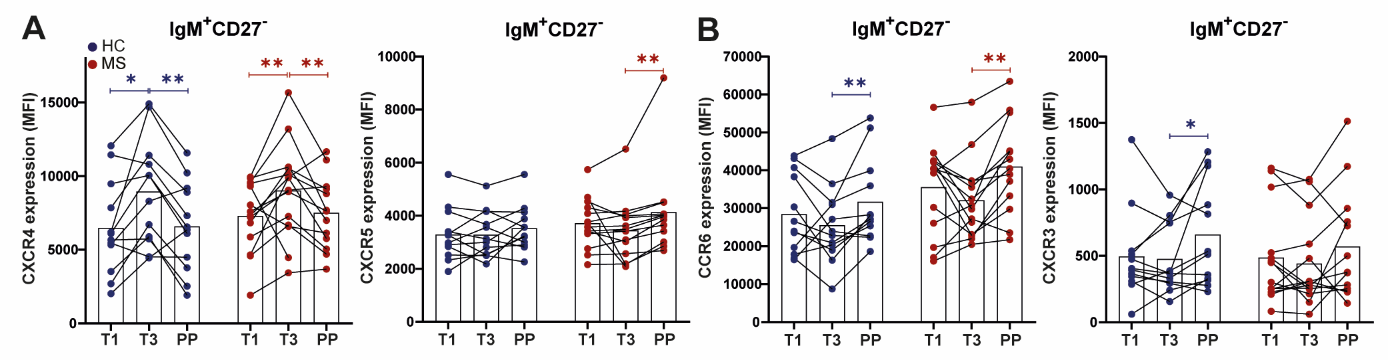
**

**Supplementary figure 3: Chemokine expression levels on naive mature B cells of HC and MS patients**

Surface expression of dark zone-associated CXCR4 and light zone-associated CXCR5, CCR6 and CXCR3 on IgM^+^CD27^-^ naive B cells in the first trimester (T1), third trimester (T3) and postpartum period (PP) for 12 HC (blue) and 13 MS patients without a postpartum relapse (red). Wilcoxon signed-rank test was performed to compare the different gestational periods. p<0.05, ** p<0.01.


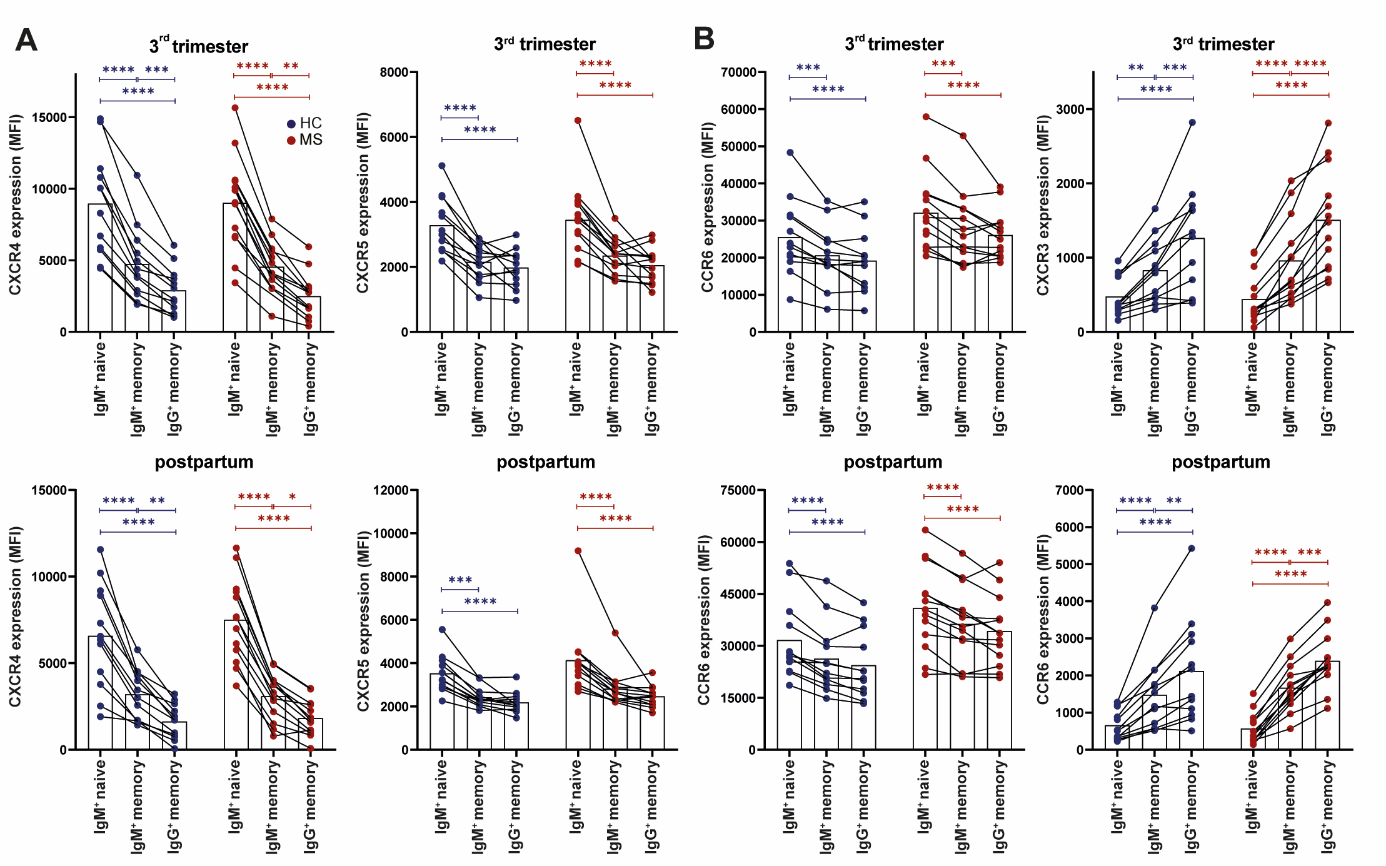


**Supplementary figure 4: Chemokine expression levels on naive mature, IgM^+^ and IgG^+^ memory B cells in the third trimester and early after delivery.**

Surface expression levels of dark zone-associated CXCR4 and light zone-associated CXCR5, CCR6 and CXCR3 were compared between IgM^+^ naive, IgM^+^ memory and IgG^+^ memory B cells from third trimester and early postpartum samples from 12 HC (blue) and 13 MS patients without a postpartum relapse (red). Two-way ANOVA was performed to compare the different B-cell subsets within a group. * p<0.05, ** p<0.01, *** p<0.001, **** p<0.0001.


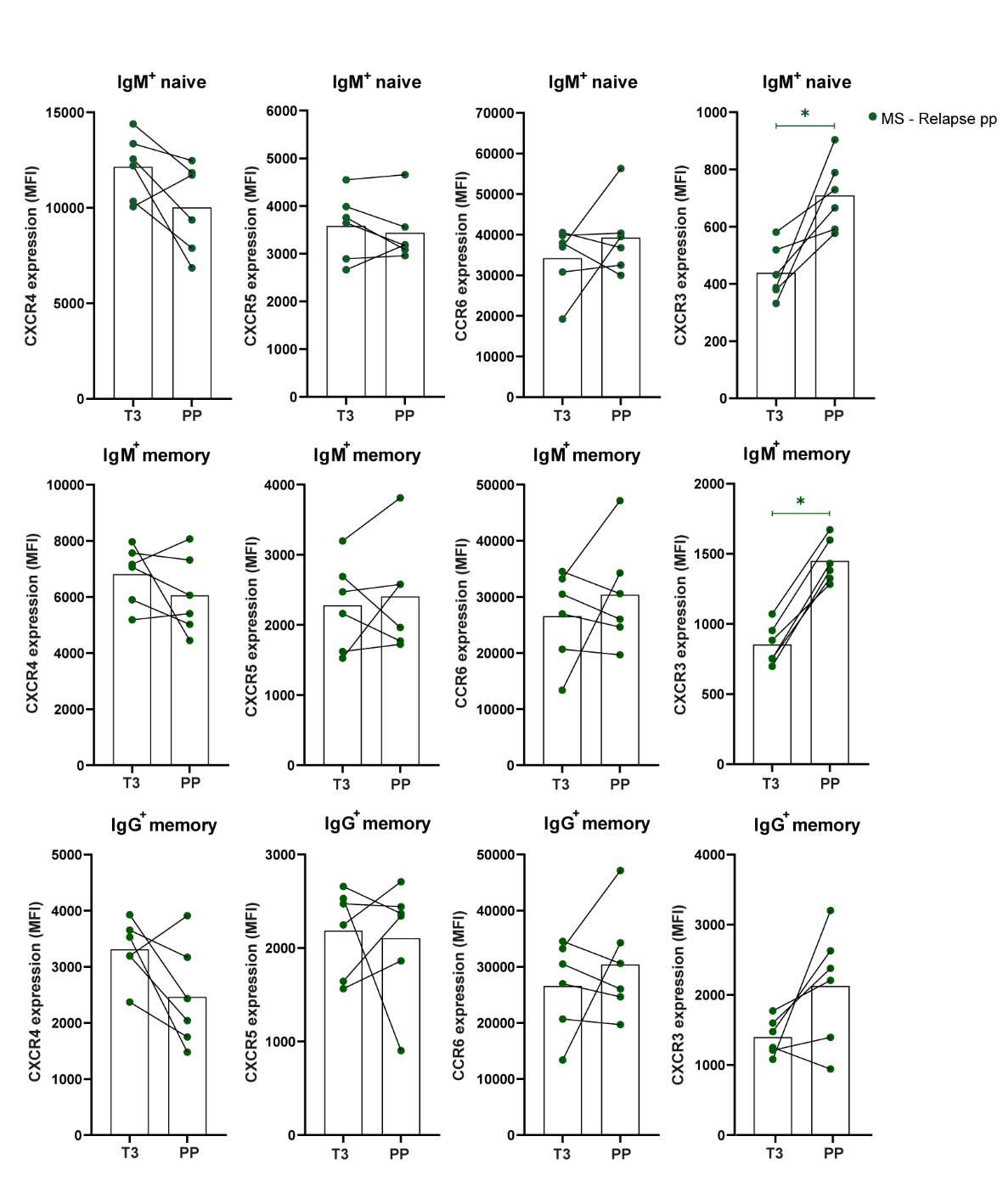


**Supplementary figure 5: Chemokine expression levels on naive mature, IgM^+^ and IgG^+^ memory B cells in the third trimester and early after delivery of MS patients experiencing a postpartum relapse**

Surface expression levels of dark zone-associated CXCR4 and light zone-associated CXCR5, CCR6 and CXCR3 on IgM^+^ naive, IgM^+^ memory and IgG^+^ memory B cells in the first trimester, third trimester (T3) and postpartum period (PP) for MS patients with a postpartum relapse (n = 6). Wilcoxon signed-rank test was performed to compare the groups. * p<0.05.
